# 3D-bioprinted tracheal reconstruction: an overview

**DOI:** 10.1186/s42234-019-0031-1

**Published:** 2019-09-17

**Authors:** Lidia Frejo, Daniel A. Grande

**Affiliations:** 1 Orthopaedic Research Laboratory, The Feinstein Institutes for Medical Research, 350 Community Drive, Manhasset, NY 11030 USA; 2 Division of Otolaryngology and Communicative Disorders-Pediatric Otolaryngology, Long Island Jewish Medical Center New Hyde Park, New York, USA; 3 Donald and Barbara Zucker School of Medicine at Hofstra/Northwell, Hempstead, New York, USA

**Keywords:** 3D-bioprinting, Tracheal reconstruction, Biomaterials

## Abstract

Congenital tracheomalacia and tracheal stenosis are commonly seen in premature infants. In adulthood, are typically related with chronic obstructive pulmonary disease, and can occur secondarily from tracheostomy, prolong intubation, trauma, infection and tumors. Both conditions are life-threatening when not managed properly. There are still some surgical limitations for certain pathologies, however tissue engineering is a promising approach to treat massive airway dysfunctions. 3D-bioprinting have contributed to current preclinical and clinical efforts in airway reconstruction. Several strategies have been used to overcome the difficulty of airway reconstruction such as scaffold materials, construct designs, cellular types, biologic components, hydrogels and animal models used in tracheal reconstruction. Nevertheless, additional long-term in vivo studies need to be performed to assess the efficacy and safety of tissue-engineered tracheal grafts in terms of mechanical properties, behavior and, the possibility of further stenosis development.

## Background

Tracheomalacia is defined as a soft cartilaginous sustenance of the trachea which can lead to the collapse and narrowing of the airway lumen during expiration. Tracheal stenosis is a rare condition where there is a narrowing of the trachea that causes breathing problems. Congenital forms of tracheomalacia and tracheal stenosis are more commonly seen in premature infants and are associated with severe symptoms (Kugler & Stanzel, 2014). Adult malacia and stenosis are typically related with chronic obstructive pulmonary disease, and can occur secondarily from tracheostomy, tumors, infection, prolonged intubation, trauma, and from external compression by vascular structures (Chan et al., 2019). Both conditions are life-threatening for the patients (Chan et al., 2019; Wain Jr., 2009). In mild-to-moderate tracheomalacia, observation or continuous positive airway pressure therapy might be effective; yet, if these fail and the malacia is severe, surgery is frequently indicated (Carden et al., 2005). Tracheal resection and re-anastomosis have existed as a solution since late nineteenth century, but it was contraindicated for stenotic segments longer than 5 cm in adults and 2 cm in children for the risk of excess tension (Ho & Koltai, 2008). Recently, slide tracheoplasty has improved outcomes significantly for inoperable patients (Wang et al., 2016; Basta et al., 2015). However, performing such a procedure comes at a sacrifice of tracheal length for tracheal inner diameter.

An innovative solution to overcoming these difficulties is the usage of artificial substitutes to replace long-segment narrowed trachea. Several studies have turned to grafting technologies to overcome the clinical needs facing tracheal repair. Nevertheless, donor-site morbidity, erosion, infection, the requirement of immunosuppression in a cancer patient, as well as exceedingly complex laboratory and surgical techniques have prevented widespread clinical use (Fabre et al., 2013; Crowley et al., 2015; Friedman & Mayer, 1992; Propst et al., 2011).

Tissue engineering is a promising approach to treat massive airway dysfunctions such as tracheomalacia or stenosis. With advances in 3D-printing techniques, many studies have combined the two in order to improve the technologies that are available for tracheal surgeries. Tissue engineering and 3D-printing techniques it is possible to design a customized tracheal model with a morphology that is suitable for the patient and that supports the force to maintain the tissue engineered trachea (TET) shape (Sing et al., 2017). The ideal combination of tissue engineering with 3D-printing should be biocompatible, biodegradable (having an appropriate degradation rate), non-immunogenic, non-toxic, with low cost, readily available, have an appropriate degradation rate and a long shelf life (Law et al., 2016).

**Fig. 1:**
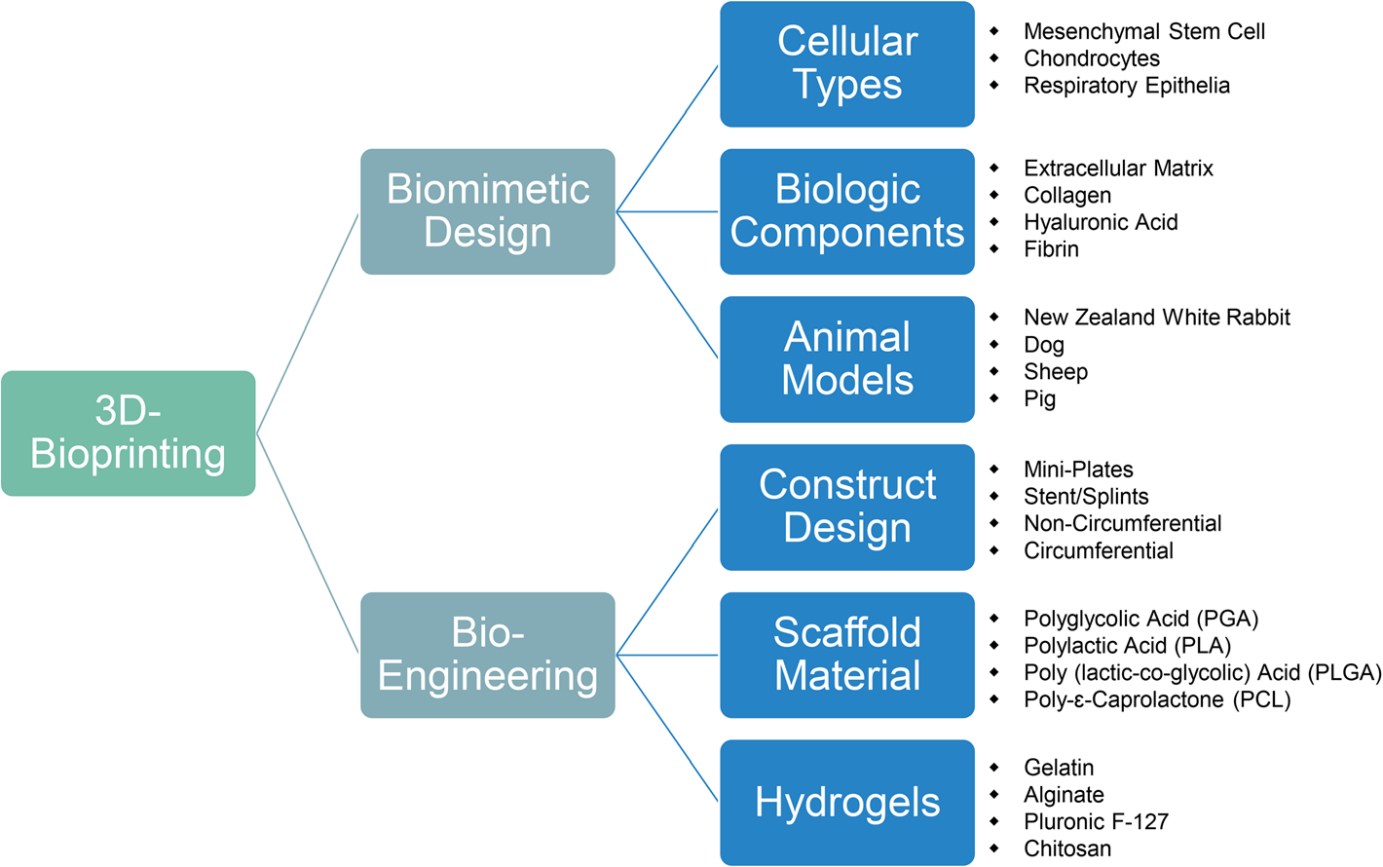
Schematic summary of all the aspects mentioned in this review

Bioprinting is at the vertex of the latest technological advances in 3D printing technologies. This specialty merges electronics (scanners, printers) and biology (organismal/architectural, cellular, protein/compounds) in order to restore form and/or function in injury or disease and is considered a type of bioelectronic medicine. This field is rapidly advancing and herein we focus on the construct design, type of material, use of cells, type of species studied, method of analysis and the need of extended in vivo test (Fig. 1).

## Main text

### Scaffold material

Several authors have previously used a variety of non-resorbable biomaterials in different experimental models. A main concern with non-resorbable biomaterials is their potential for inducing a chronic inflammatory response with granulation tissue and extrusion (Yener et al., 2010; Daneshi et al., 2010; Gaafar et al., 2008). On the contrary, resorbable biomaterials, like different thermoplastic polymers: polyglycolic acid (PGA), poly(lactic-co-glycolic) acid (PLGA), polylactic acid (PLA), and poly-ɛ-caprolactone (PCL) used in 3D printing have similar properties to the tracheal cartilage; consequently, various attempts are being made to apply these materials to artificial trachea research (Chang et al., 2014).

PLA was one of the most popular materials used in 3D printing because it is already approved by the Food and Drug Administration for various uses (Athanasiou et al., 1996). Its mechanical properties help maintain the structure of the construct (Goldstein et al., 2015). PGA fibers usage in tracheal replacement was first reported in 1994 (Vacanti et al., 1994). Both PGA and PLGA have been typically used in engineered tracheal scaffold because of their high porosity, which can induce cell infiltration and neovascularization and can be absorbed at a relatively accurate time. However, due to its short absorption time and inadequate mechanical strength, it has been difficult to use for long-term therapeutic effect (Kojima et al., 2002; Rotter et al., 2005; Wu et al., 2007). Other studies have used mixtures of PLA-PGA (Long et al., 2001) or PLA-PLGA (Klein et al., 2005), yet the findings showed that the addition of other types of materials to PLA composites caused a decrease in the mechanical features of the composite used (Tappa et al., 2019). On the other hand, PCL has been used as a cartilage scaffold material because of its mechanical features, non-toxic degradation products, good biocompatibility and slow biodegradation. Properties such as low porosity, long absorption time and strength are superior to PGA, therefore PCL has long-term applicability. Some studies have also shown that low porosity promotes chondrogenesis (Karageorgiou & Kaplan, 2005). Considering its comparatively low melting point, PCL is easily printed and can be used with commercial desktop 3D printers as confirmed by Kaye et al. (Kaye et al., 2019).

### Construct design

Several repair methods have been reported in order to avoid resection and reanastomosis. We can categorize 4 extensive groups for the construct design in tracheal surgeries: a) Miniplates b) Stent/Splint c) Non-circumferential reconstruction and d) Circumferential reconstruction (Chan et al., 2019).

#### Miniplates

Bioabsorbable miniplates have been used safely as an alternative to autologous cartilage grafts for single stage laryngotracheal reconstruction (LTR) (Sprecher, 2010). Sprecher reported good results performing anterior split laryngotracheoplasty on 10 patients with subglottic stenosis using a resorbable PLA miniplate to keep the cricoid expansion without cartilage grafting. All patients showed fully mucosalized tracheal wall and there were no postoperative complications (Sprecher, 2010). Javia and Zur used a commercially available resorbable miniplate as an external lateral support in 7 pediatric patients undergoing LTR surgery who had unexpected airway malacia in addition to stenosis. Six children were successfully decannulated within 3 months with no further complications (Javia & Zur, 2012). Recently, Goldstein et al. demonstrated the effectivity of 3D-printed PLA miniplates seeded with mature chondrocytes and collagen gel implanting them successfully in New Zealand white rabbits (NZW) for a total of 12 weeks (Goldstein et al., 2015).

#### Stent/splint

Airway stents or splints have been widely used for the treatment of tracheobronchial pathologies since late twentieth century (Liu et al., 2011). A silicone-based T-Tube developed by Montgomery in the 1960’s is one of the earliest airway stents (Montgomery, 1965).

Silicone and metallic stents have been the most used in treating airway diseases. However, complications including prosthesis migration, granulation formation, sputum retention, stent fracture have been reported (Martinez-Ballarin et al., 1996; Lemaire et al., 2005; Chung et al., 2008; Chin et al., 2008).

Stents made of resorbable materials have advantages over silicone and metallic stents because they degrade over time and their removal is not necessary. Biodegradable stents made of polydioxanone; a material used in sutures, has been reported recently for the relief of anastomotic stenosis. Lischke et al. reported their first clinical application in 6 post-LT patients where 5 of them were in good clinical condition after 4 years’ follow-up (Lischke et al., 2011). Later, Fechner et al. reported a larger case series with a total of 11 stents (Fuehner et al., 2013).

Three-dimensional printing includes the opportunity of designing customized airway stents. Therefore, patients’ computerized axial tomography (CT) scan can be used to create quality cross-section images that are then stacked to create a 3D image of the scanned trachea to build personalized TET (Do et al., 2015). PCL has become the most used biomaterial in this approach and has already moved from animal studies to human patients (Huang et al., 2016; Zopf et al., 2014; Morrison et al., 2014). Thus, Hollister et al. begun to implement design control for scaffold-based tissue engineering approaches founded on 3D-printing (Hollister et al., 2015). Using this design, Les et al. reported 15 pediatric subjects with severe tracheobronchomalacia, receiving successfully 29 3D-printed and patient-specific splints on their trachea (Les et al., 2019).

#### Non-circumferential reconstruction

Different shapes and sizes are used in this approach, from small rectangular pieces to 2 cm long 270° tracheal reconstruction. Within this group, it appears less granulation and stenosis are present. PCL has become the most used material in this approach. Various studies describing the usage of small rectangular shapes show no evidence of stenosis and minimal granulation tissue over time. Histology displays regeneration of ciliated epithelium and neuro-vascularization on luminal surface (Park et al., 2018a; Park et al., 2012; Kwon et al., 2014; Jang et al., 2014). When larger defects were created and repaired on larger animals, results are variable. Townsend et al., used 15x25mm PCL implants in sheep. The animals had to be euthanized prior to the end of the study due to respiratory distress secondary to tracheal narrowing at the reconstruction site (Townsend et al., 2018). On the other hand, Rehmani et al. reconstructed a 40 × 16 mm defect in 7 pigs with a PCL implant covered with an extracellular matrix and found that 5 pigs had well-sized tracheal lumen with minimal stenosis and granulation tissue after 3 months (Rehmani et al., 2017).

#### Circumferential reconstruction

Circumferential reconstruction is the most difficult, with longer defects being more challenging than shorter ones since they can induce more granulation tissue causing stenosis and respiratory difficulties. Granulation formation is a common complication tracheal surgery. This process is mediated by a wide range of cellular reactions such as infection, inflammation, tissue necrosis and immunological rejection (Lee et al., 2011; Nicolli et al., 2016). The granulation and stenosis seen in the scaffold segment after transplantation might be associated to the lack of protective epithelial layer along with an inflammatory reaction. Therefore, different biologic components were added to the construct in order to accelerate cell growth and migration to minimize granulation tissue formation.

Gao et al. 3D-printed a tracheal scaffold with biodegradable material with a chondrocyte suspension and implanted the construct in the subcutaneous tissue of nude mice to overcome the inflammatory process. To evaluate the feasibility of repairing whole segment tracheal defects, replacement surgery of rabbits’ native trachea by the construct was performed (Gao et al., 2017). Lee et al. assessed the use of immunosuppressive therapy after tracheal replacement however, they resolved there were no beneficial effects (Lee et al., 2017).

### Cellular types, biologic components and hydrogels

Given the development of bio-printing technology, living cells can be added to a hydrogel for printing, and cells, such as chondrocytes or respiratory epithelia which play an important role in the tracheal structure, as well as mesenchymal stem cells (MSC), can be printed together in the production of artificial tracheas (Boland et al., 2006). A wide range of natural and synthetic components have been tested to promote cartilage formation for tracheal regeneration. These materials include collagen, extracellular matrix (ECM) containing molecules from the collagen family, elastic fibers, glycosoaminoglycans (GAG) and proteoglycans, and adhesive glycoproteins, gelatin, chitosan, hyaluronic acid, alginate, fibrin glue, DegraPol, acellular cartilage tissue matrices or Pluronic F-127 (Kwon et al., 2014; Jang et al., 2014; Rosso et al., 2004) that can be used alone or combined. While naturally derived scaffolds have a countless advantage in biocompatibility and neovascularization, an implant that is only made of natural materials lacks proper mechanical features and structural integrity for tracheal reconstruction (Schwarz et al., 2012). Hence, combining a naturally derived scaffold with a synthetic polymer could be key for tissue-engineered tracheal reconstruction.

Park et al. constructed a multilayered scaffold using PCL and alginate hydrogel with auricular cartilage and nasal epithelia. The artificial tracheas were transplanted into 15 rabbits for up to 12 months. Several rabbits died from respiratory symptoms. From the surviving rabbits, narrowed tracheas due to granulation were found. Their trachea seemed to be effective in respiratory epithelia regeneration but not in cartilage formation (Park et al., 2019). Recently, Park et al., have created a tissue-engineered PCL graft by stratifying tracheal mucosa decellularized extracellular matrix (tmdECM) collagen hydrogel together with human inferior turbinate mesenchymal stromal cell (hTMSC) sheets. After 2 months, there was a complete regeneration of the luminal surface of the construct. Some granulation could be observed at the transplantation site but no severe complications were observed (Park et al., 2018b).

Chondrocytes are frequently used in tracheal tissue engineering for cartilage regeneration, but the limited supply of autologous chondrocytes and the difficulty in maintaining their phenotype during in vitro culture have thwarted their wider application. Kim et al. described a successful partial tracheal reconstruction using a fibrin/hyaluronan hydrogel seeded with chondrocytes, but neo-cartilage regeneration was barely seen in their results (Kim et al., 2010).

At the same time, bone marrow MSC (bMSC), as multipotent stem cells, can differentiate into chondrogenic phenotypes with proper stimulation. Co-culture of bMSCs with chondrocytes stimulates and improves the differentiation of bMSCs when both are in the same environment. In previous studies, rabbit MSC co-cultured with chondrocytes in hydrogel constructs were found to undergo differentiation into chondrocytes (Kang et al., 2012). Liu et al. used a co-culture system to mimic the articular chondrogenic configuration in subcutaneous environments (Liu et al., 2010). Tsao et al. developed a ring-shaped tracheal scaffold using either PGLA or PCL stented with silicone rod and seeded with bMSC and chondrocytes for 7 days before implantation in NZW rabbits. Analysis of the biochemical and mechanical characteristics established that the PCL scaffold with co-culture cells seeding exhibited the optimal chondrogenesis with acceptable rigidity to maintain the cylindrical shape and luminal patency (Tsao et al., 2014). Recently, Bae et al. used a similar approach co-culturing chondrogenic-differentiated bMSC and respiratory epithelial cells in one scaffold. Neocartilage formation, neo-epithelization and neo-vascularization could be observed (Bae et al., 2018). Chang et al. 3D-printed a 10x10mm half-pipe-shaped PCL scaffold coated with rabbit MSC seeded in human-derived fibrin and then implanted for 8 weeks. Neo-cartilage was enough to keep the scaffold shape (Chang et al., 2014).

### Animal models

In the 1995, Delaere et al. developed a rabbit model for orthotopic tracheal transplantation after a period of heterotopic revascularization in the lateral thoracic fascia (Delaere et al., 1995). This animal model has become the most commonly used animal model as authors argue it is the ideal model for LTR. Rabbits have a long cervical trachea that is easily accessible and resembles a human trachea in structure and size to that of an infant. Rabbits have a more diverse genetic background compared to rodents making a better approximation to human’s genetic diversity (Bosze & Houdebine, 2010; Graur et al., 1996). They are simple to manage, extensively available, and cost-effective for interventional studies (Park et al., 2018a). Nevertheless, dog, sheep, and pig models might be better to reproduce the size of teenagers with adult sized tracheas, but these large animals are difficult to manage post-operatively and are more expensive (Zopf et al., 2014; Townsend et al., 2018; Rehmani et al., 2017).

## Conclusion

**Fig. 2:**
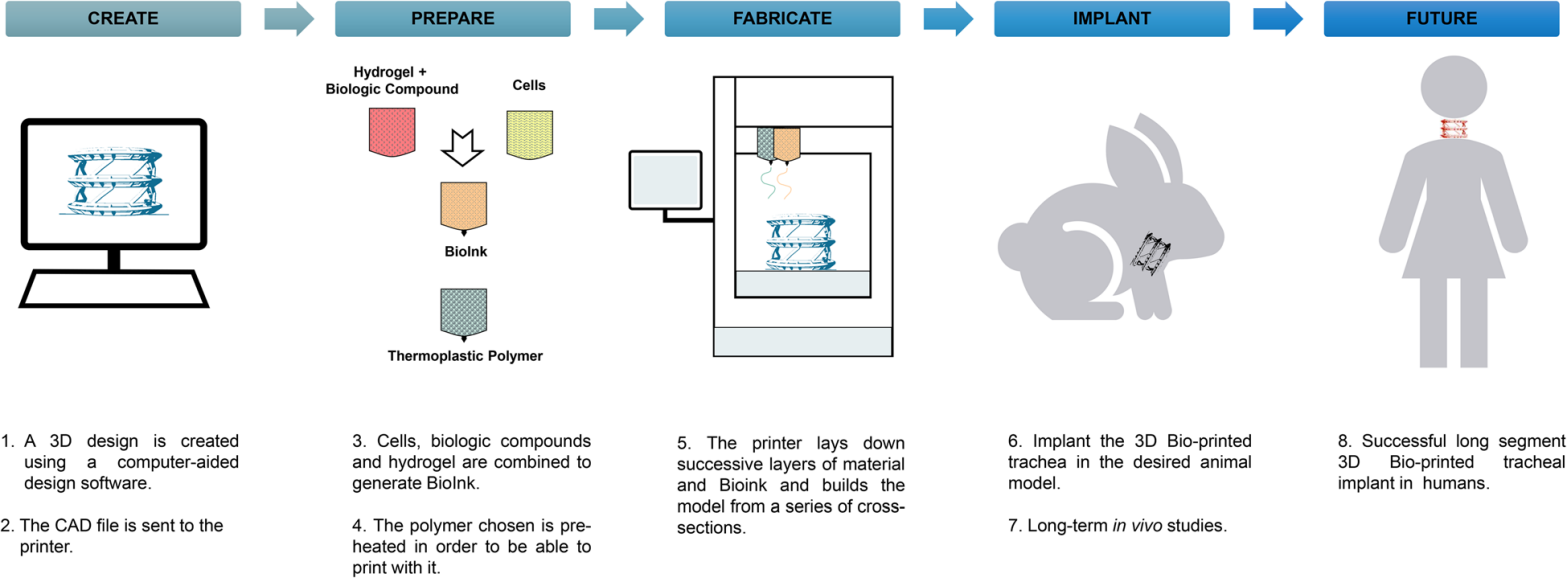
Pipeline and future directions in 3D-bioprinted tracheal reconstruction

Bioprinting is at the vertex of the latest technological advances in 3D printing technologies. This specialty merges biology and electronics to restore form and/or function in injury or disease (Fig. 2). In this review we surveyed the most commonly used materials, designs, cellular types, biologic components, hydrogels and animal models used in 3D-bioprinting tracheal reconstruction.

Material-wise, PCL is a rising biodegradable material that can be used for tracheal surgery. Although its usefulness on its own, the combination of PCL with cells seeded in naturally derived materials improves its effectiveness. Besides, rabbit is the most widely used animal model used in TET reconstruction as its size and shape likens the infant human trachea. Further in vivo studies need to be assessed to determine the best animal model for adult-size trachea.

Finally, additional long-term in vivo studies need to be performed to assess the efficacy and safety of the TET grafts. In order to preserve the function and enhance long-term survival and grafting rates, vascularization and epithelization of the graft need yet to be widely considered when dealing with airway reconstruction.

## Data Availability

Not applicable.

## Abbreviations

bMSC: Bone marrow MSC
ECM: extracelular matrix
hTMSC: human inferior turbinate mesenchymal stromal cell
LTR: laryngotracheal reconstruction
MSC: Mesenchimal Stem Cells
NZW rabbits: New Zealand White rabbits
PCL: poly-ɛ-caprolactone
PGA: polyglycolic acid
PLA: polylactic acid
PLGA: poly(lactic-co-glycolic) acid
TET: tissue engineered trachea
tmdECM: tracheal mucosa decellularized extracellular matrix
